# Effects of Cognitive Behavioral Therapy for Diet on Postprandial Glucose and Pregnancy Outcomes in Gestational Diabetes Mellitus: Multicenter Randomized Controlled Trial

**DOI:** 10.2196/71075

**Published:** 2025-07-29

**Authors:** Ying Pan, Jia Tang, Bing Lu, Ming Kuang, Mengjie Zhao, Hongying Liu, Shao Zhong

**Affiliations:** 1 Department of General Medicine, Kunshan Hospital Affiliated to Jiangsu University, Suzhou, China; 2 Hangzhou Kang Ming Information Technology Co., Ltd, 301 Building 12, Haichuang Park 998 Wenyi West Road, Hangzhou, Zhejiang Province, 310000, China, 86 021 64045531; 3 Department of Endocrinology, Kunshan Hospital Affiliated to Jiangsu University, Suzhou, China

**Keywords:** digital dietary intervention, cognitive behavioral therapy, glycemic qualification rate, pregnancy outcomes, gestational diabetes mellitus, mobile phone

## Abstract

**Background:**

Gestational diabetes mellitus (GDM) is associated with an elevated risk of adverse maternal and neonatal outcomes. Dietary management is a cornerstone of GDM treatment due to its beneficial effects on metabolic control. However, suboptimal adherence to dietary recommendations has diminished its potential benefits in achieving optimal glycemic outcomes. Cognitive behavioral therapy (CBT)–based interventions have emerged as a promising approach to enhance dietary compliance and glycemic control in patients with GDM.

**Objective:**

This study aims to investigate the effects of a CBT-based digital dietary intervention on glycemic control and pregnancy outcomes in patients with GDM.

**Methods:**

The intervention group received standard care plus a digital dietary intervention based on CBT principles, delivered via a customized WeChat (Tencent Inc) mini program. This intervention included structured dietary education and behavioral strategies focused on appropriate food selection and meal sequencing. The control group received standard care alone. The primary outcome was the glycemic qualification rate, and secondary outcomes included fasting blood glucose, postprandial blood glucose (PBG), General Self-Efficacy Scale scores, and incidence of macrosomia. Self-monitored blood glucose data were collected and analyzed at biweekly follow-up visits from enrollment until delivery.

**Results:**

Of the 200 participants, 171 completed the study. The average age was 31.2 (SD 4) years, and the average gestational age at enrollment was 26.3 (SD 1.6) weeks. Baseline HbA_1c_ levels were similar between groups (5.2% vs 5.1%; *P=.*97). The glycemic qualification rate was significantly higher in the intervention group than in the control group at follow-up 3 (mean 87.9%, SD 14.9% vs 81.9%, SD 17.8%; *P*=.02), follow-up 4 (mean 91.0%, SD 9.9% vs 87.2%, SD 14.4 %; *P*=.04), follow-up 5 (mean 94.0%, SD 7.4% vs 91.5%, SD 9.5%; *P*=.04), and follow-up 6 (mean 94.3%, SD 6.7% vs 91.8%, SD 8.9%). PBG levels were significantly lower in the intervention group after lunch (1 h: mean 5.9, SD 0.7 vs 6.0, SD 0.7 mmol/L; *P*=.0 2 h2h: 5.1, SD 0.7 vs 5.3, SD 0.8 mmol/L; *P*=.03) and dinner (1 h: mean 6.0, SD 0.5 vs 6.2, SD 0.6; 2 h: 5.5, SD 0.7 vs 5.7, SD 0.8 mmol/L). However, no significant differences were observed in fasting blood glucose or PBG after breakfast between the groups. The intervention group showed significantly higher General Self-Efficacy Scale scores than the control group (mean 195.4, SD 6.9 vs 192.9, SD 5.8). The incidence of macrosomia was significantly lower in the intervention group than in the control group (5% vs 15%; *P*=.04).

**Conclusions:**

The findings of this randomized controlled trial suggest that a CBT-based digital dietary intervention can significantly enhance glycemic control, particularly PBG levels, and may contribute to improved pregnancy outcomes with a reduced incidence of macrosomia in women with GDM.

## Introduction

### Background

Gestational diabetes mellitus (GDM) is defined as any degree of glucose intolerance with onset or first recognition during pregnancy [1]. GDM represents one of the most prevalent medical complications associated with pregnancy. The overall prevalence of GDM in China has been documented at 14.9%, based on epidemiological surveillance data [2]. According to the International Diabetes Federation, GDM complicates over 14% of pregnancies worldwide, with an estimated 18 million live births annually exposed to maternal hyperglycemia [3]. GDM is associated with a spectrum of adverse maternal and neonatal outcomes, including pre-eclampsia, fetal malformations, cesarean delivery, premature delivery, macrosomia, and neonatal hypoglycemia during the perinatal period. Furthermore, both mothers and their offspring are at increased risk for developing type 2 diabetes mellitus later in life [4-6].

GDM arises from a complex interplay of hormonal, metabolic, and genetic factors. During pregnancy, placental hormones (eg, human placental lactogen) induce physiological insulin resistance to redirect glucose to the fetus, which may exceed maternal pancreatic β-cell compensatory capacity in predisposed individuals [7]. Key nondietary risk factors include prepregnancy overweight or obesity, advanced maternal age (>35 y), and a family history of type 2 diabetes [8]. Additionally, accumulating evidence has suggested that dietary intake before and during pregnancy is associated with the risk of developing GDM [9]. Current clinical guidelines advocate for dietary management as the first-line intervention before the initiation of pharmacological therapy due to its established efficacy in improving metabolic outcomes in patients with GDM [10]. Dietary approaches such as low glycemic index diets, low glycemic load (GL) diets, and fiber-enriched diets have been shown to significantly reduce fasting plasma glucose, 2-hour postprandial glucose levels, and HbA_1c_, in addition to improving neonatal health indicators [11]. Moreover, low GL diets have been associated with reduced cesarean delivery risk [11].

Despite the demonstrated benefits, the implementation of dietary interventions remains challenging, largely due to the requirement for sustained nutritional counseling and behavioral education to facilitate long-term dietary changes [12-14].

### Evidence Before This Study

According to current clinical guidelines, glycemic control in patients with GDM should be managed proactively to prevent associated maternal and fetal complications [15]. Instant messaging apps, routine email reminders, and telephone consultations have demonstrated potential in supporting the management of GDM by enhancing patient management and compliance [16]. However, these conventional interventions often demand considerable time and resource commitments from health care professionals, thereby limiting scalability and long-term sustainability [17].

Recent advancements in digital health have led to the development of innovative digital interventions aimed at improving self-management and patient education among individuals with GDM. These digital solutions have shown promising outcomes in achieving better glycemic control [18,19]. Nevertheless, some studies have reported comparable efficacy between conventional and new digital approaches, suggesting that the added value of digital intervention may vary depending on the content and implementation strategy [20-22].

Cognitive behavioral therapy (CBT) is a structured, evidence-based psychotherapeutic approach that aims to identify and modify maladaptive thought patterns and behaviors, thereby facilitating sustainable behavioral changes [23]. Digital CBT refers to the delivery of CBT through digital platforms and includes components such as assessment, formulation, treatment, training, and supervision [24]. Digital CBT has been proposed as a promising tool for enhancing chronic disease management, with mounting evidence supporting its effectiveness in promoting health-related behavioral modifications [25-28]. Meta-analyses of randomized controlled trials have demonstrated that CBT-based interventions significantly reduced HbA_1c_ in patients with diabetes [29]. The observed improvements in glycemic control have been attributed to enhanced adherence to lifestyle recommendations and reductions in psychological distress, including symptoms of depression and anxiety [30].

Despite these encouraging findings, there remains a notable paucity of research investigating the application of CBT-based interventions for dietary behavior modification in patients with GDM [13,31,32]. We designed and implemented a smartphone-based digital therapeutic platform for GDM management, integrating CBT-guided dietary interventions with the goal of improving glycemic control in women with GDM.

### Objectives

This multicenter, randomized, controlled, open-label clinical trial aimed to evaluate the effects of a CBT-based dietary intervention on glycemic control and pregnancy outcomes in patients with GDM.

## Methods

### Study Design

The study was conducted at four hospitals in Kunshan, China: the First People’s Hospital of Kunshan, the Second People’s Hospital of Kunshan, the Fourth People’s Hospital of Kunshan, and the Jinxi People’s Hospital of Kunshan. The enrollment period spanned from July 11, 2021, to June 30, 2022, with study completion on December 31, 2023. The trial concluded once all enrolled participants completed the intervention and follow-up assessments.

### Ethical Considerations

The study protocol was approved by the Medical Ethics Committee of Kunshan Hospital affiliated with Jiangsu University (approval 2021-03-004-H01). The study was prospectively registered in the Chinese Clinical Trials Registry (ChiCTR2100048527) on July 9, 2021. No amendments were made to the study protocol after registration. Participation in the study was entirely voluntary. No financial or material compensation was offered to participants. Written informed consent was obtained from all participants prior to enrollment. Participants were informed of their right to withdraw at any time without any impact on their medical care. They were also provided with detailed information regarding the study objectives, potential risks, dietary information, and the importance of adhering to the study protocol. All personal data were anonymized, and each participant was assigned a unique study identification number for data collection and analysis purposes.

### Recruitment

Eligible participants were pregnant women who underwent an oral glucose tolerance test (OGTT) between 24 and 28 weeks of gestation at an endocrinology clinic. GDM diagnosis was established by endocrinologists according to standard diagnostic criteria based on the results of the OGTT.

All participants underwent a standard 75-gram OGTT between 24 and 28 weeks of gestation, as recommended by the International Association of Diabetes and Pregnancy Study Groups and endorsed by the World Health Organization (WHO) [33,34]. Participants were instructed to fast overnight for at least 8 hours prior to testing. Venous blood samples were collected at three time points: fasting (0 min), 1 hour, and 2 hours after the oral ingestion of a 75-gram glucose solution.

GDM was diagnosed if one or more of the following plasma glucose values met or exceeded the International Association of Diabetes and Pregnancy Study Group diagnostic thresholds [33]: fasting plasma glucose ≥5.1 mmol/L (92 mg/dL); 1-hour postglucose load ≥10.0 mmol/L (180 mg/dL); 2-hour postglucose load ≥8.5 mmol/L (153 mg/dL).

The study investigators were responsible for recruiting participants. Eligible patients with GDM were identified in the endocrinology clinic and invited in person by the investigators to participate in the study. The investigators provided detailed information about the study’s purpose, procedures, and ethical considerations. After providing written informed consent, patients were screened according to the study protocol, and those who met the inclusion criteria were enrolled. A total of 200 participants were enrolled.

Inclusion criteria were as follows: (1) age 18‐45 years, (2) completion of OGTT at 24‐28 weeks of gestation, (3) diagnosis of GDM without prior initiation of pharmacological therapy, (4) ability to use a smartphone and basic literacy and numeracy skills in Chinese, and (5) willingness to provide informed consent. Exclusion criteria were as follows: (1) a diagnosis of diabetes prior to pregnancy, (2) a history of GDM in a previous pregnancy, (3) the presence of severe mental illness or cognitive impairment, and (4) any conditions that could interfere with participation in the study.

### Randomization and Blinding

In this trial, participants were randomly assigned in a 1:1 ratio to the intervention or control group using a centralized, computerized randomization system. Investigators accessed the system using secure login credentials. A unique random number generated at the time of patient registration was used to track participants throughout the study. The study was not blinded due to the nature of the intervention.

### Interventions

In this study, a digital intervention was developed and delivered via a customized WeChat (Tencent Inc) mini program (Multimedia Appendix 1). The content of the intervention was designed by a team of experienced endocrinologists from Kunshan Hospital Affiliated with Jiangsu University based on their clinical expertise in the management of GDM. The technical development and customization of the WeChat mini program were carried out by Hangzhou Kangming Information Technology Co., Ltd, based on the specifications provided by the clinical team. Prior to clinical implementation, the WeChat mini program underwent usability and functionality testing with a pilot group of 30 users. Participants were recruited from the same endocrinology clinic population. The 30 users were randomly selected from eligible patients with GDM using a computer-generated randomization list. They were asked to use the mini program for 1 week, after which structured feedback was collected through a standardized usability questionnaire and semistructured interviews. Feedback focused on ease of navigation, content clarity, and user satisfaction. Based on this input, the interface design and instructional content were refined. Following these revisions and quality assurance review, the WeChat mini program was approved for use in the subsequent clinical study.

Both the intervention and control groups received standardized care in accordance with the Guidelines for the Diagnosis and Treatment of Hyperglycemia in Pregnancy (2022) [35,36]. Participants were taught basic information about GDM and how to perform self-management, including how to conduct self-monitoring of blood glucose (SMBG), what the target blood glucose values are, and how to keep a lifestyle diary. All participants were provided with blood glucose meters, test strips, and patient diaries to record SMBG data using capillary whole blood. Participants used the Accu-Chek Guide glucometer and test strips (Roche Diabetes Care GmbH) to measure fingertip capillary blood glucose levels. In cases where glycemic targets were not achieved through lifestyle modifications, pharmacological treatment was considered by the clinical care team. Use of medications for other chronic, nondiabetic conditions was permitted. During the treatment period, the use of hypoglycemic agents, functional foods, and dietary supplements intended to lower blood glucose levels was prohibited.

Participants in the intervention group received a 12-week CBT-based digital dietary program via a customized WeChat mini program. The intervention consisted of personalized courses, delivered three times weekly (30 min per session), and focused on the principles of proper food choices and meal sequence skills. The intervention was divided into cognitive and behavioral components [23].

Cognitive components included: (1) knowledge about the relationship between GDM pathophysiology and diet [29], (2) knowledge about the identification of low glycemic index and low-GL foods, (3) knowledge about food-exchange methods [30], and (4) knowledge about differentiation of foods based on macronutrient content (eg, water, fiber, protein, fat, and carbohydrate; Multimedia Appendix 2).

Behavioral components included: (1) skill in using the calorie counting method and calculating daily caloric requirements; (2) skill in applying the food combining method and mixing different foods; (3) skill in implementing specific meal sequence strategies, including starting meals with water or fiber-rich foods, followed by protein-rich or fat-rich foods, and concluding with carbohydrate-rich foods.

Participants were oriented to the WeChat mini program’s functionality at the outset of the intervention. The control group received only standardized care without access to the digital intervention.

### Measurements and Outcomes

All data were collected and managed using an electronic data capture (EDC) system. After participants signed the informed consent forms, baseline demographics and clinical characteristics, including age, gestational age, and immediate family history of diabetes, were obtained by the investigators. The primary outcome of the study was the change in the glycemic qualification rate (GQR) in both groups during the follow-up period. GQR was defined as the percentage of fingerstick blood glucose values that met the predefined glycemic targets over a specific period [37]. Secondary outcomes included individual SMBG values, scores on the General Self-Efficacy Scale (GSES) at weeks 4, 8, and 12, and the incidence of macrosomia assessed at 42 days postpartum.

All patients in the study were instructed to perform SMBG according to the study protocol. The procedure for blood glucose testing, data collection, and data monitoring was consistent for all patients. Participants used the glucometer and test strips to measure fingertip capillary blood glucose levels. Results were manually recorded by patients in paper-based logbooks. During each follow-up visit, the investigators reviewed and collected these logbooks and transcribed the data into the EDC. Participants in both groups were instructed to monitor blood glucose at the following time points: fasting, postbreakfast (1 and 2 h), postlunch (1 and 2 h), and postdinner (1 and 2 h), on two nonconsecutive days each week. A total of 14 monitored glucose values were obtained per week. Each follow-up period encompassed 2 weeks, during which 28 SMBG readings were expected per participant. Follow-up visits were conducted biweekly, coinciding with routine prenatal care appointments.

GQR was calculated using the following formula: GQR (%)=(number of blood glucose values within target range/28)×100. The glycemic targets for pregnant women were defined as follows: fasting blood glucose (FBG) <5.3 mmol/L, 1-hour postprandial blood glucose (PBG) (postbreakfast, postlunch, and postdinner blood glucose) <7.8 mmol/L, and 2-hour PBG (postbreakfast, postlunch, and postdinner blood glucose) <6.7 mmol/L. FBG and PBG values were obtained from SMBG reports.

GSES, a validated instrument used to assess patients’ general self-efficacy, was administered in paper format (Multimedia Appendix 3) [38]. All patients completed these forms independently. During each follow-up, these completed forms were reviewed and entered into the EDC by the study investigators.

After all patients were enrolled in the study, the investigators documented their expected delivery dates and subsequently recorded the actual delivery dates during follow-up. At 42 days postpartum, participants were contacted via telephone by the investigators to report the newborn’s birth weight. This information was verified using the official birth records of the newborns. Macrosomia was defined as a birth weight of ≥4000 grams.

### Statistical Analysis

According to previous research, the baseline GQR was approximately 65% in patients with GDM [31]. The sample size calculation was based on a pilot trial, which indicated a 65% GQR in the control group and an anticipated mean difference of 20% between the two groups [31]. To detect this difference at a 5% (two-sided) significance level with a power of 80%, a minimum of 86 participants per group was required. Considering an estimated 20% dropout rate, the final sample size was increased to 100 participants per group.

Continuous variables with a normal distribution were presented as mean (SD), and group comparisons were conducted using independent samples 2-tailed tests. Categorical variables were summarized as frequencies and percentages, with between-group comparisons performed using the chi-square test. For nonnormally distributed continuous variables, data were presented as medians, and group comparisons were performed using the Kruskal-Wallis test. The distribution of glucose values over the 12-week study period was visualized using violin plots and statistically assessed using the Kruskal-Wallis test. A *P* value of less than .05 was considered indicative of statistical significance.

The primary outcome, GQR, was treated as a normally distributed continuous variable. To compare GQR between the intervention and control groups over time, we initially conducted independent samples 2-tailed tests at each follow-up time point. To enhance interpretability, we also calculated absolute between-group differences in GQR along with 95% CIs.

To further address potential baseline imbalance and reduce bias from regression to the mean, we conducted a supplementary ANCOVA for follow-up time points 2 through 6. In each ANCOVA model, group assignment (intervention vs control) was the independent variable, and GQR at follow-up 1 (wk 1‐2) was included as a covariate to adjust for baseline differences. Adjusted group means (estimated marginal means), absolute between-group differences, 95% CI, and *P* values were reported.

The secondary outcomes, including SMBG values and GSES scores, were also assumed to follow normal distributions and were analyzed using independent samples 2-tailed tests.

## Results

### Study Participants

**Figure 1.**
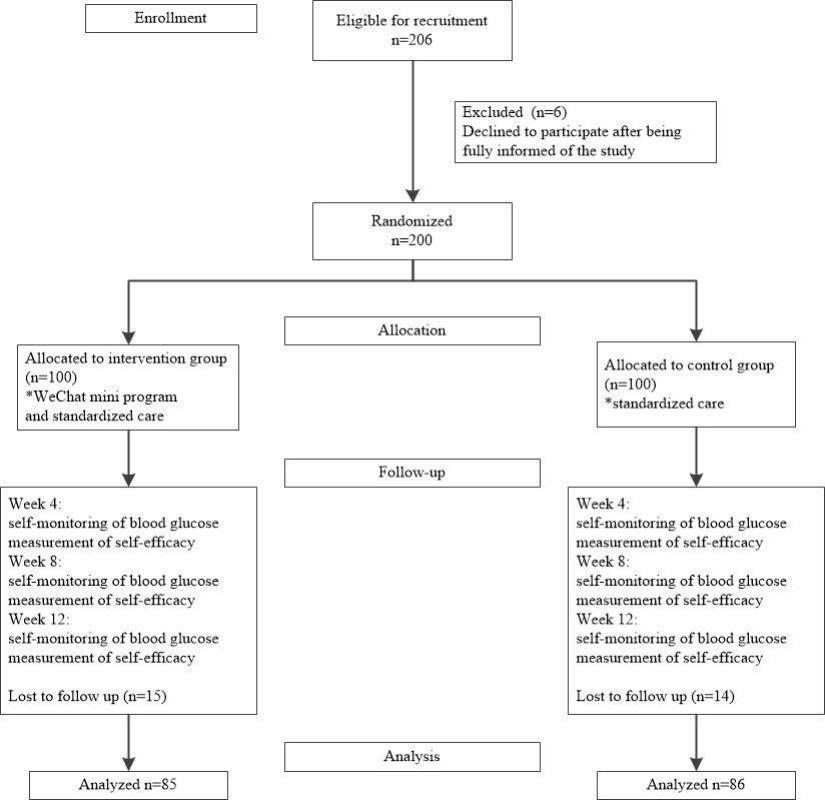
Flow diagram of the study.

A total of 200 participants diagnosed with GDM were randomly assigned to either the intervention group (n=100) or the control group (n=100). Of these, 171 participants completed the follow-up assessments (85 in the intervention group and 86 in the control group), while 29 participants were excluded due to study withdrawal or loss to follow-up. The study flowchart is shown in Figure 1.

### Baseline Characteristics

Participants ranged in age from 27 to 36 years and were enrolled at 24 to 28 weeks of gestation. Baseline clinical characteristics included 2-hour OGTT values ranging from 8.6 to 13.0 mmol/L and glycated hemoglobin (HbA_1c_) levels between 4.8% and 5.6%. Additionally, 34% (68/200) of participants reported a family history of diabetes. There were no statistically significant differences between the intervention and control groups in baseline demographic and clinical characteristics, as detailed in Table 1.

**Table 1.** Baseline demographic and clinical characteristics.

Variable	Overall (n=171)	Control (n=86)	Intervention (n=85)	*P* value
Age (years), mean (SD)	31.2 (4.0)	31.6 (4.2)	30.8 (3.9)	.19
Gestational age (weeks), mean (SD)	26.3 (1.6)	26.2 (1.7)	26.4 (1.4)	.41
Height (cm), mean (SD)	160.6 (4.8)	161.2 (4.7)	160.1 (4.8)	.16
Weight (kg), mean (SD)	67.6 (8.5)	67.8 (8.3)	67.4 (8.8)	.76
2-hour OGTT^a^ (mmol/L), mean (SD)	10.8 (2.1)	10.6 (2.2)	10.9 (2.0)	.47
FBG^b^ (mmol/L), mean (SD)	5.0 (0.5)	4.9 (0.6)	5.1 (0.5)	.58
HbA_1c_ (%), mean (SD)	5.2 (0.4)	5.3 (0.2)	5.1 (0.4)	.97
Immediate family history of diabetes, n (%)	68 (40)	31 (36)	37 (44)	.40

a OGTT: oral glucose tolerance test.

b FBG: fasting blood glucose.

### Primary Outcome

**Figure 2.**
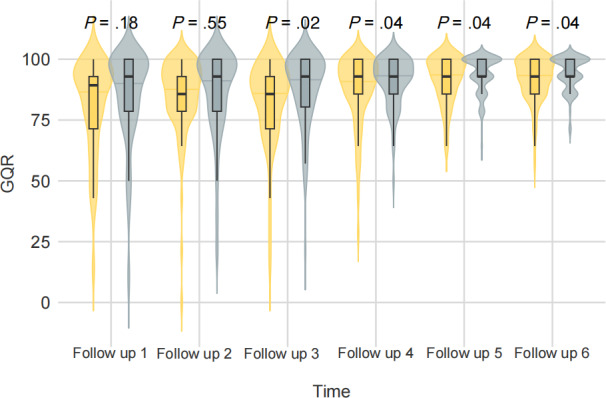
GQR distribution in the two groups during the follow-up period. The horizontal axis displays different follow-ups (2 wk as one follow-up). The vertical axis represents the value of GQR (%). The yellow shadow area represents the whole distribution of the control group, whereas the blue shadow area represents the whole distribution of the intervention group. Each half of the violin plot contains a boxplot of the data. The middle short horizontal lines are medians, and the black rectangles represent IQR. The 2-tailed test was used to compare differences between groups. GQR: glycemic qualification rate.

Following the 12-week intervention period, participants in the intervention group exhibited significantly improved glycemic control compared to those in the control group (Table 2 and Figure 2). Specifically, the GQR in the intervention group was significantly higher than in the control group at follow-ups 3 to 6 (all *P*<.05). In contrast, no statistically significant differences in GQR were observed at follow-ups 1 and 2 (all *P*>.05). Between-group differences ranged from 2.5% to 6%, and all 95% CIs from follow-up 3 (weeks 5‐6) to follow-up 6 (weeks 11‐12) excluded zero, suggesting consistent improvements in glycemic control attributable to the intervention.

**Table 2.** GQR^a^ values in the two groups during the follow-up period.

Time point	Overall (%; n=171), mean (SD)	Control (%; n=86), mean (SD)	Intervention (%; n=85), mean (SD)	Between-group difference (95% CI)	*P* value
Follow-up 1	82.7 (19.9)	80.7 (20.0)	84.7 (19.6)	4.0 (−1.4 to 9.3)	.18
Follow-up 2	85.4 (17.3)	84.6 (16.6)	86.1 (18.0)	1.5 (−3.4 to 6.5)	.55
Follow-up 3	84.9 (16.7)	81.9 (17.8)	87.9 (14.9)	6.0 (1.1 to 10.9)	.02
Follow-up 4	89.1 (12.5)	87.2 (14.4)	91.0 (9.9)	3.8 (0.2 to 7.4)	.04
Follow-up 5	92.7 (8.6)	91.5 (9.5)	94.0 (7.4)	2.5 (0.1 to 5.0)	.04
Follow-up 6	93.0 (8.0)	91.8 (8.9)	94.3 (6.7)	2.5 (0.1 to 5.0)	.04

a GQR: glycemic qualification rate.

In supplementary ANCOVA analysis adjusting for baseline GQR at follow-up 1 (weeks 1‐2), the intervention group demonstrated a significantly higher adjusted GQR at follow-up 3 (weeks 5‐6) compared with the control group (*P*=.049). Although group differences at later time points did not reach statistical significance after adjustment, the intervention group consistently showed numerically higher adjusted GQR values throughout the follow-up period (Table S1 in Multimedia Appendix 4). These findings suggest that the intervention had an early measurable impact on glycemic control, with a sustained though attenuated trend in subsequent weeks.

### Secondary Outcomes

Following the 12-week intervention, PBG levels after lunch and dinner in the intervention group were significantly lower than those in the control group. Specifically, the mean 1-hour and 2-hour postlunch PBG values were 5.9 (SD 0.7) mmol/L versus 6.0 (SD 0.7) mmol/L (*P*=.04) and 5.1 (SD 0.7) mmol/L versus 5.3 (SD 0.8) mmol/L (*P*=.03), respectively. Similarly, the mean 1-hour and 2-hour postdinner PBG values were 6.0 (SD 0.5) mmol/L versus 6.2 (SD 0.6) mmol/L (*P*=.03) and 5.5 (SD 0.7) mmol/L versus 5.7 (SD 0.8) mmol/L (*P*=.03), respectively. However, there were no significant differences in FBG (*P*=.84) and postbreakfast PBG levels between the two groups (*P*=.13 and *P*=.69; Tables3  4). Notably, antidiabetic drugs were not used in either group during the follow-up period of this study.

**Table 3.** Glucose values in two groups at baseline.

Glucose values (baseline)		Overall (n=171), mean (SD)	Control (n=86), mean (SD)	Intervention (n=85), mean (SD)	*P* value
FBG^a^ (mmol/L)		4.8 (0.5)	4.8 (0.6)	4.8 (0.5)	.55
PBG^b^ (mmol/L)					
1 hour after breakfast		7.3 (1.1)	7.4 (1.1)	7.2 (1.0)	.30
2 hours after breakfast		5.8 (0.9)	5.8 (1.0)	5.7 (0.8)	.66
1 hour after lunch		6.6 (1.2)	6.7 (1.3)	6.5 (1.0)	.46
2 hours after lunch		6.0 (0.8)	6.0 (0.9)	6.0 (0.7)	.93
1 hour after dinner		6.6 (1.0)	6.5 (1.1)	6.7 (0.9)	.35
2 hours after dinner		6.0 (0.9)	6.0 (1.0)	6.0 (0.8)	.82

a FBG: fasting blood glucose.

b PBG: postprandial blood glucose.

**Table 4.** Glucose values in two groups at week 12.

Glucose values (week 12)		Overall (n=171), mean (SD)	Control (n=86), mean (SD)	Intervention (n=85), mean (SD)	*P* value
FBG (mmol/L)		4.4 (0.4)	4.4 (0.4)	4.3 (0.3)	.84
PBG (mmol/L)					
1 hour after breakfast		6.4 (1.3)	6.5 (1.4)	6.2 (1.2)	.13
2 hours after breakfast		5.3 (0.9)	5.3 (0.9)	5.3 (0.9)	.69
1 hour after lunch		6.0 (0.7)	6.0 (0.7)	5.9 (0.7)	.04
2 hours after lunch		5.2 (0.7)	5.3 (0.8)	5.1 (0.7)	.03
1 hour after dinner		6.2 (0.6)	6.2 (0.6)	6.0 (0.5)	.03
2 hours after dinner		5.6 (0.8)	5.7 (0.8)	5.5 (0.7)	.03

At both weeks 8 and 12, GSES scores in the intervention group were significantly higher than those in the control group. At week 8, the mean GSES score was 191.6 (SD 7.0) in the intervention group versus 189.8 (SD 6.5) in the control group (*P*=.004). At week 12, the mean GSES score increased to 195.4 (SD 6.9) in the intervention group compared to 192.9 (SD 5.8) in the control group (*P*=.003; Table 5).

**Table 5.** GSES^a^ scores in two groups during the follow-up period^b^.

GSES	Overall (n=171), mean (SD)	Control (n=86), mean (SD)	Intervention (n=85), mean (SD)	*P* value
Baseline	186.3 (7.1)	185.7 (7.0)	186.9 (7.1)	.26
Week 4	187.0 (7.2)	186.9 (7.1)	187.1 (7.2)	.31
Week 8	190.7 (6.8)	189.8 (6.5)	191.6 (7.0)	.004
Week 12	194.2 (6.5)	192.9 (5.8)	195.4 (6.9)	.003

a GSES: General Self-Efficacy Scale.

b GSES scores are the sum of the 20 items in the self-efficacy scale.

The incidence of macrosomia (neonatal birth weight ≥4000 g) was significantly lower in the intervention group than in the control group (n=4, 5% vs n=13, 15%; *P*=.04). However, there was no statistically significant difference in mean neonatal birth weight between the two groups (3163.7, SD 651.3 g in the intervention group vs 3389.3, SD 575.9 g in the control group; *P*=.07).

## Discussion

### Principal Findings

This randomized controlled trial demonstrated that a CBT-based digital dietary intervention significantly improved GQR, reduced PBG, enhanced GSES, and lowered the incidence of macrosomia among pregnant women with GDM compared to conventional care. Specifically, the GQR in the intervention group was significantly higher at most follow-up time points (follow-ups 3, 4, 5, and 6). PBG values were significantly lower in the intervention group than in the control group (1 h and 2 h after lunch and dinner). GSES scores at weeks 8 and 12 were significantly higher in the intervention group than in the control group. Importantly, a lower rate of macrosomia was observed in the intervention group than in the control group.

### Comparison to Prior Work

Chronic disease management, particularly for GDM, relies heavily on lifestyle modifications, including diet and physical activity. CBT has been increasingly recognized as an effective strategy in diabetes care, particularly for promoting behavioral changes that enhance adherence to treatment and improve metabolic outcomes [39,40]. Prior studies have demonstrated the benefits of CBT-based dietary interventions in improving dietary self-management among women with GDM by improving cognition and guiding behavior change [41-43]. However, optimal dietary regimens for GDM remain undefined [44-46]. This study showed that a digitally delivered CBT-based dietary intervention can maintain high GQR and promote sustained glycemic control in patients with GDM.

Digital health tools have gained attention for their potential to extend self-management support while reducing the need for frequent in-person visits [47]. In this study, the digital intervention was education-focused and designed to enhance dietary awareness, problem-solving, and self-regulation through CBT principles. The significant increase in GQR in the intervention group demonstrated that CBT plays an important role in ongoing self-management. The steady improvement in GSES during the intervention period, particularly from weeks 8 to 12, suggests that repeated exposure to CBT content reinforced participants’ confidence in managing their condition. This is consistent with previous findings that self-efficacy is a critical determinant of glycemic control [10].

In GDM, postprandial hyperglycemia is more prevalent and clinically relevant than fasting hyperglycemia [48]. Persistent postprandial hyperglycemia can lead to endothelial cell dysfunction, excessive inflammation, increased cell adhesion, and cell proliferation [49,50]. Pedersen proposed that maternal hyperglycemia results in fetal hyperglycemia and, consequently, hypertrophy of fetal islet tissue with insulin hypersecretion. The high rate of glucose utilization by fetuses increases the likelihood of neonatal abnormalities [51]. In this study, although PBG values were modestly but significantly lower in the intervention group than in the control group, especially after lunch and dinner, there was no significant difference in FBG or postbreakfast PBG between the two groups. This may be attributable to dietary patterns specific to the local population or to a higher degree of insulin resistance in the morning due to elevated levels of cortisol and growth hormone [52].

### Disparity Between Glycemic Control Improvements and Birth Outcomes

An intriguing observation in this study is that despite only a modest (approximately 3%) difference in GQR at week 12, the intervention group exhibited a markedly lower incidence of macrosomia (5% vs 15%). While we did not perform a direct correlation analysis between GQR and birth weight, this discrepancy raises important questions about the interpretation of glycemic control metrics and their relationship to clinical outcomes.

Several studies have highlighted that even small improvements in glycemic control, particularly reductions in postprandial glucose, can have disproportionately large effects on fetal outcomes [47,53]. The Hyperglycemia and Adverse Pregnancy Outcome study found a continuous relationship between maternal glucose levels and the risk of macrosomia, even within what are considered “normal” ranges [54]. Additionally, digital interventions may influence outcomes through mechanisms not fully captured by GQR alone, such as optimizing meal timing, reducing glycemic variability, and fostering more consistent behavior over time [37,55,56]. The educational content and CBT framework may have supported the adoption of sustained dietary patterns conducive to fetal growth moderation.

Nevertheless, without a direct assessment of dietary intake, behavioral adherence, or insulin dynamics, this finding must be interpreted with caution. Future studies should evaluate these mediating factors more comprehensively and explore the potential for nonlinear or threshold effects between glycemic improvement and fetal outcomes, which may be attributable to dietary patterns.

### Strengths and Implications

This study offers valuable insights into the feasibility and effectiveness of a CBT-based dietary intervention in real-world prenatal care. As dietary management remains the cornerstone of GDM therapy, our findings suggest that digital CBT tools can serve as an effective adjunct to standard care. This digitally delivered CBT-based dietary intervention differs from conventional models. It required no direct oversight by health care professionals, emphasizing patient autonomy and potentially offering a scalable solution for resource-limited settings.

The study also demonstrated the positive impact of digital interventions on self-efficacy in women with GDM. Improvements in self-efficacy suggest that digital psychological support can enhance patient engagement and may help bridge gaps in conventional care.

### Limitations

Despite the promising findings, certain limitations should be acknowledged. (1) Scope of digitalization: the mini program was used solely for educational purposes, while participants continued to monitor blood glucose using paper records. Integration of real-time glucose monitoring and feedback could enhance intervention effectiveness; (2) adherence measurement: individual adherence to the mini program and completion rates of CBT content were not assessed. Without objective engagement data, the dose-response relationship between intervention exposure and outcomes remains unclear; (3) dietary intake assessment: changes in dietary behavior were inferred but not directly measured. Future studies should use validated dietary recall tools or digital food diaries to assess how CBT strategies influence actual food choices and nutrient intake; (4) glycemic control: both groups achieved relatively high levels of glycemic control, potentially reducing the detectable effect size and limiting generalizability; (5) sample size: although the sample size was based on power calculations, it remains modest. Future trials with larger cohorts are needed; (6) generalizability: this study was conducted exclusively in East China. Cultural dietary patterns and regional health care infrastructure may limit applicability to other populations; (7) blinding: due to the behavioral nature of the intervention, participants and researchers were not blinded, introducing potential expectancy bias; (8) biomarkers: patient-level biomarkers such as weight, insulin, cortisol, and growth hormone were not collected due to resource constraints. Their absence may have obscured some physiological effects of the intervention; (9) long-term data: follow-up ended at 12 weeks postpartum; therefore, the long-term sustainability of the intervention’s effects remains unknown.

### Conclusions and Future Directions

This randomized controlled trial suggests that a CBT-based digital dietary intervention can improve glycemic control, enhance self-efficacy, and reduce the risk of macrosomia in patients with GDM. These findings support the integration of psychological frameworks and digital tools in the dietary management of GDM.

However, the modest improvements in glycemic markers alongside a substantial reduction in macrosomia underscore the complex and potentially nonlinear relationship between behavioral interventions and clinical outcomes. Future research should focus on elucidating the behavioral, psychological, and metabolic pathways that mediate these outcomes. In particular, exploring dietary adherence, glycemic variability, and long-term maternal outcomes will be essential to validate and optimize digital CBT interventions for women with GDM.

## Supplementary material

10.2196/71075 Multimedia Appendix 1 WeChat mini program Demo.

10.2196/71075 Multimedia Appendix 2 Cognitive components.

10.2196/71075 Multimedia Appendix 3 General self-efficacy scale.

10.2196/71075 Multimedia Appendix 4 ANCOVA analysis.

10.2196/71075 Checklist 1 CONSORT-eHEALTH checklist (V 1.6.1).

## Abbreviations

CBT: cognitive behavioral therapy
EDC: electronic data capture
FBG: fasting blood glucose
GDM: gestational diabetes mellitus
GL: glycemic load
GQR: glycemic qualification rate
GSES: General Self-Efficacy Scale
OGTT: oral glucose tolerance tests
PBG: postprandial blood glucose
SMBG: self-monitored blood glucose
WHO: the World Health Organization
