# Supplementary figures and images for: Effects of Cognitive Behavioral Therapy for Diet on Postprandial Glucose and Pregnancy Outcomes in Gestational Diabetes Mellitus: Multicenter Randomized Controlled Trial
Source: J Med Internet Res. 2025 Jul 29;27:e71075. doi: 10.2196/71075 (PMC12306952; doi:10.2196/71075)

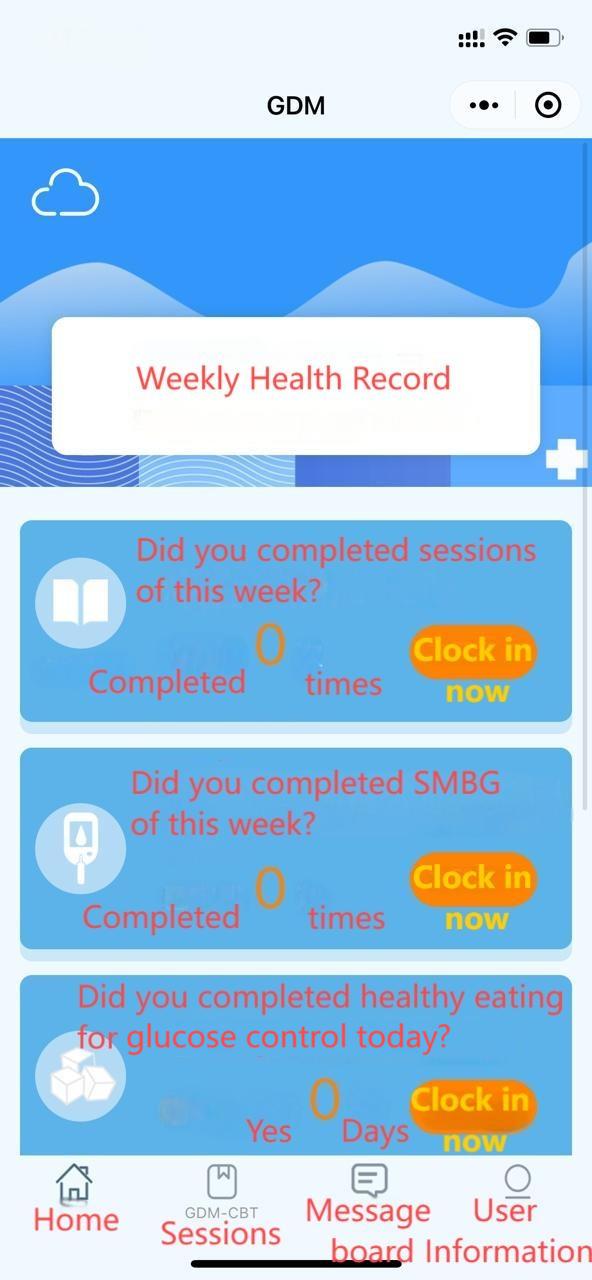

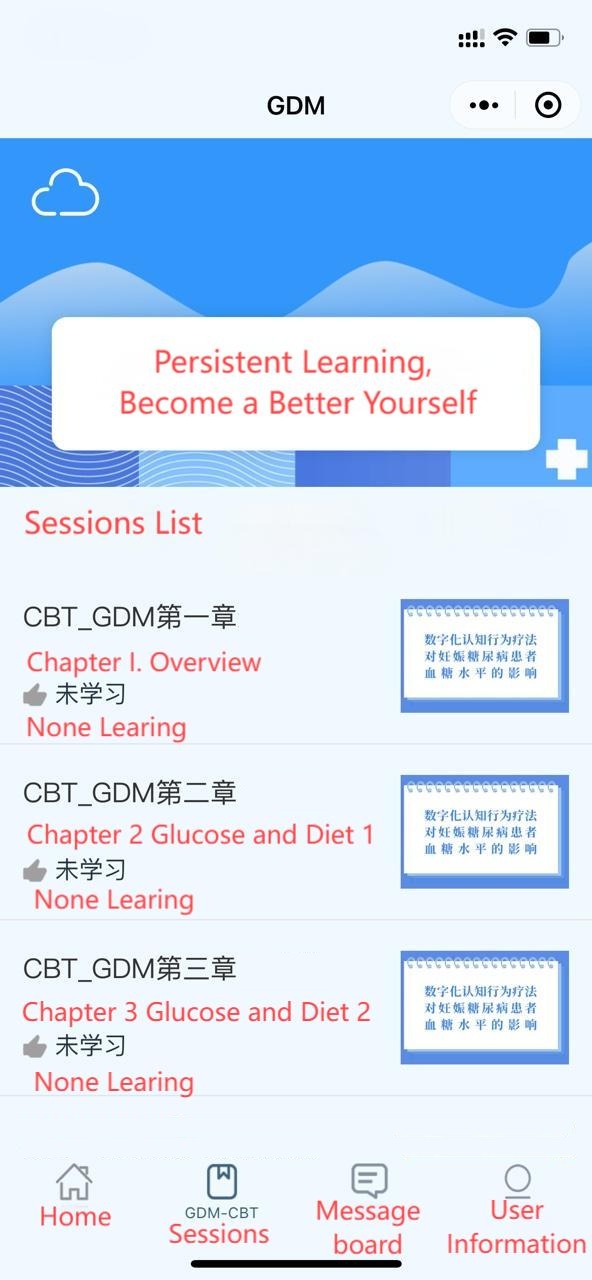


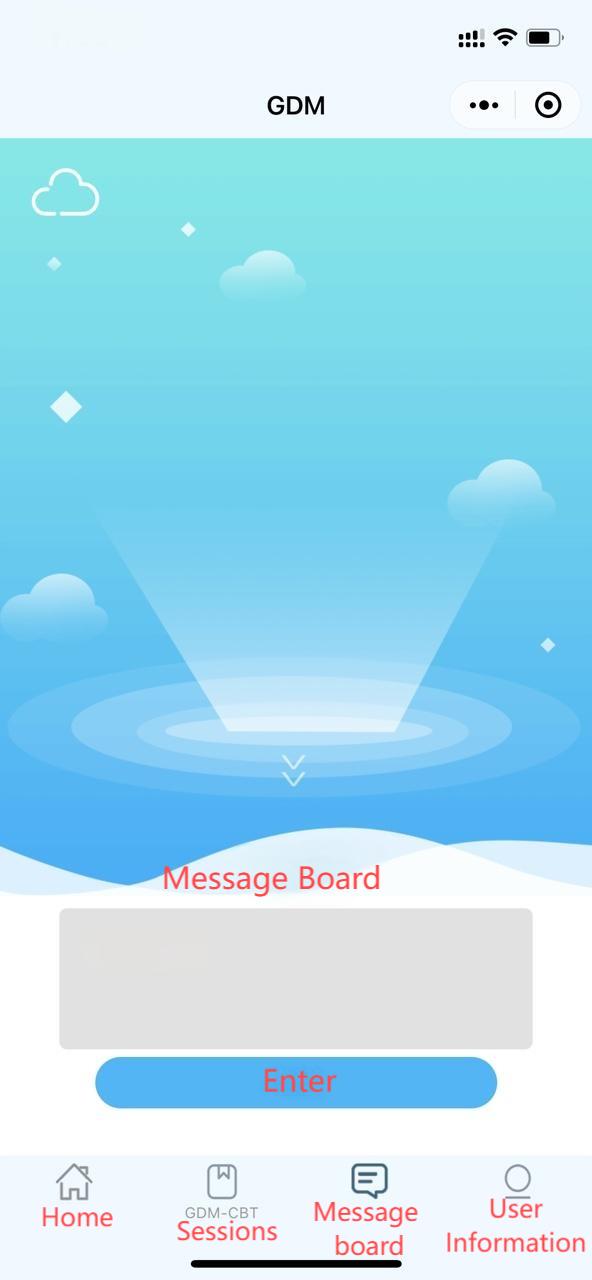

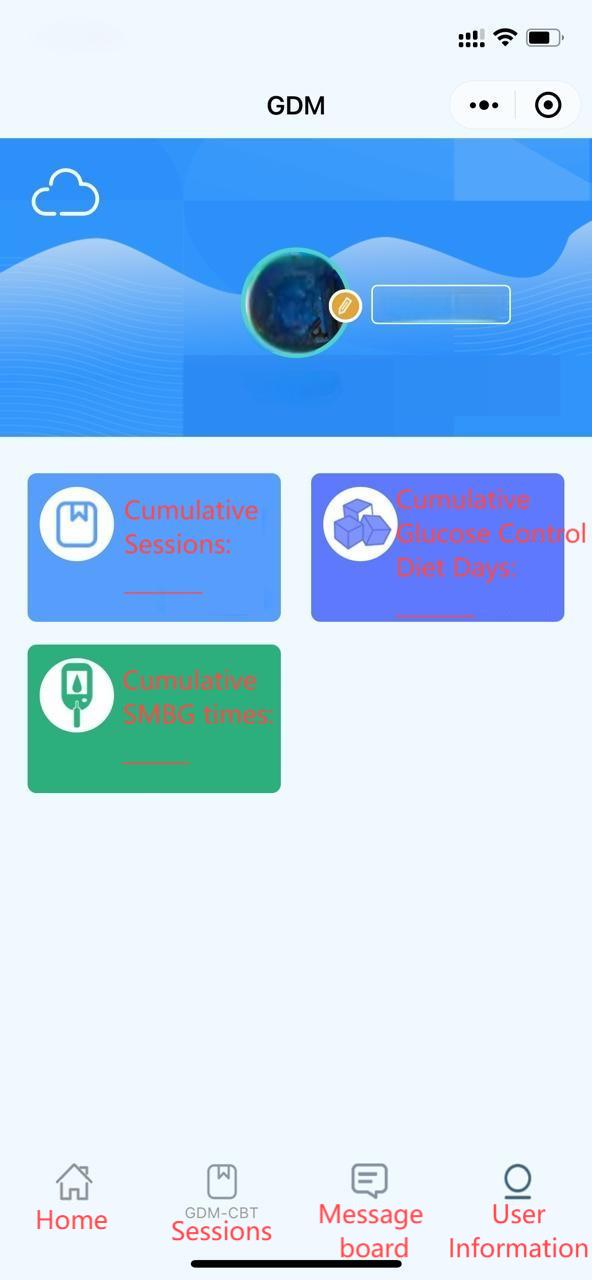

Supplement: Multimedia Appendix 1 [file jmir-v27-e71075-s001.docx]
