# Supplementary material for: Effects of Cognitive Behavioral Therapy for Diet on Postprandial Glucose and Pregnancy Outcomes in Gestational Diabetes Mellitus: Multicenter Randomized Controlled Trial
Source: J Med Internet Res. 2025 Jul 29;27:e71075. doi: 10.2196/71075 (PMC12306952; doi:10.2196/71075)
Supplement: Multimedia Appendix 2 [file jmir-v27-e71075-s002.docx]

Low-GI foods^a^

| Food | GI | Food | GI | Food | GI | Food | GI |
| --- | --- | --- | --- | --- | --- | --- | --- |
| Chocolate | 49.0 | Yogurt | 48.0 | Buckwheat | 54.0 | Yam | 51.0 |
| Fructose | 23.0 | Snow konjac | 17.0 | Sweet potato | 54.0 | Bread | 50.0 |
| Lentils | 38.0 | non-fat milk | 32.0 | Pear | 36.0 | Apple | 36.0 |
| Chickpeas | 33.0 | Milk | 27.6 | Peaches | 28.0 | Macaroni | 45.0 |
| Tofu | 31.9 | Whole milk | 27.0 | Teak | 25.0 | Taro | 47.7 |
| Mung bean | 27.2 | Peanuts | 14.0 | Plums | 24.0 | Wheat | 41.0 |
| Green beans | 27.0 | Plantains | 53.0 | Cherries | 22.0 | Noodles | 37.0 |
| Dried tofu | 23.7 | Kiwi | 52.0 | Grape | 43.0 | Lotus root | 32.6 |
| Soybean | 18.0 | Banana | 52.0 | Tangerine | 43.0 | Barley | 25.0 |
| Fava bean | 16.9 | Rice Bran | 19.0 | Cornmeal porridge | 51.8 | Black rice porridge | 42.3 |

^a^GI(glycemic index): GI＜55, Low-GI Foods; GI＞70, High-GI Foods

Low-GL foods^a^

| Food | GL | Food | GL | Food | GL |
| --- | --- | --- | --- | --- | --- |
| instant noodle | 7.2 | lotus Seed | 5.0 | watermelon | 9.9 |
| batata starch | 7.1 | steamed taro | 5.0 | ripe banana | 8.1 |
| whole milk | 1.5 | yam | 4.4 | pineapple | 6.3 |
| pumpkin | 5.9 | mung bean | 3.8 | kiwifruit | 6.2 |
| carrot | 5.5 | green beans | 3.3 | soymilk | 4.9 |
| potato starch | 2.7 | rice noodles | 3.2 | apple | 4.4 |
| orange | 4.4 | strawberry | 4.3 | pear | 3.7 |
| grapes | 4.3 | mango | 3.9 | peach | 3.1 |
| peanut | 0.4 | grapefruit | 2.3 | skim milk | 2.6 |
| cashew | 0.4 | plain yogurt | 2.3 | onion | 1.2 |
| plum | 1.9 | cherry | 2.2 | dried bean curd | 1.3 |
| spiced lima beans | 2.5 | cellophane noodles | 5.0 | frozen bean curd | 0.8 |

^a^GL(glycemic load): GL≤10, Low-GL Foods; GL≥20, High-GL Foods

Food exchange list^a^

| Category | Per serving(g) | kJ(kcal) | Protein(g) | fat(g) | Carbohydrate(g) | main nutrients |
| --- | --- | --- | --- | --- | --- | --- |
| cereals and tubers | 25 | 376(90) | 2.0 | — | 20.0 | carbohydrate,dietary fiber |
| vegetables | 500 | 376(90) | 5.0 | — | 17.0 | vitamin, dietary fiber,  inorganic salt |
| fruits | 200 | 376(90) | 1.0 | — | 21.0 |  |
| soybeans | 25 | 376(90) | 9.0 | 4.0 | 4.0 | protein, fat |
| dairy | 160 | 376(90) | 5.0 | 5.0 | 6.0 |  |
| meat and eggs | 50 | 376(90) | 9.0 | 6.0 |  |  |
| nuts | 15 | 376(90) | 4.0 | 7.0 | 2.0 | fat, protein |
| oil | 10 | 376(90) |  | 10.0 |  |  |

^a^The food exchange method involves dividing the food items into four categories according to their source and nature, with one exchange portion determined for each type of food. The energy of each food is 376kJ, and the food exchange portions can be exchanged equally.
