# Supplementary material for: Effects of Cognitive Behavioral Therapy for Diet on Postprandial Glucose and Pregnancy Outcomes in Gestational Diabetes Mellitus: Multicenter Randomized Controlled Trial
Source: J Med Internet Res. 2025 Jul 29;27:e71075. doi: 10.2196/71075 (PMC12306952; doi:10.2196/71075)
Supplement: Multimedia Appendix 3 [file jmir-v27-e71075-s003.docx]

Self-efficacy Scale

|  | Self-rating Scores | | | | | | | | | | |
| --- | --- | --- | --- | --- | --- | --- | --- | --- | --- | --- | --- |
| 1. I am able to check my blood sugar if necessary | 0 | 1 | 2 | 3 | 4 | 5 | 6 | 7 | 8 | 9 | 10 |
| 2. I am able to correct my blood sugar when the sugar level is too high (e.g.eat different foods) | 0 | 1 | 2 | 3 | 4 | 5 | 6 | 7 | 8 | 9 | 10 |
| 3. I am able to correct my blood sugar when the sugar level is too low (e.g.eat different foods) | 0 | 1 | 2 | 3 | 4 | 5 | 6 | 7 | 8 | 9 | 10 |
| 4. I am able to choose the foods that are best for my health | 0 | 1 | 2 | 3 | 4 | 5 | 6 | 7 | 8 | 9 | 10 |
| 5. I am able to choose different foods and maintain a healthy eating plan | 0 | 1 | 2 | 3 | 4 | 5 | 6 | 7 | 8 | 9 | 10 |
| 6. I am able to control my body weight and maintain it within the ideal weight range | 0 | 1 | 2 | 3 | 4 | 5 | 6 | 7 | 8 | 9 | 10 |
| 7. I am able to examine both of my feet (e.g. for cuts or blisters) | 0 | 1 | 2 | 3 | 4 | 5 | 6 | 7 | 8 | 9 | 10 |
| 8. I am able to do enough physical activity (e.g. walking the dog, yoga, gardening, stretching exercises) | 0 | 1 | 2 | 3 | 4 | 5 | 6 | 7 | 8 | 9 | 10 |
| 9. I am able to maintain my eating plan when I am ill | 0 | 1 | 2 | 3 | 4 | 5 | 6 | 7 | 8 | 9 | 10 |
| 10. I am able to follow a healthy eating plan most of the time | 0 | 1 | 2 | 3 | 4 | 5 | 6 | 7 | 8 | 9 | 10 |
| 11. I am able to do more physical activity if the doctor advises me to do | 0 | 1 | 2 | 3 | 4 | 5 | 6 | 7 | 8 | 9 | 10 |
| 12. When doing more physical activity, I am able to adjust my eating plan | 0 | 1 | 2 | 3 | 4 | 5 | 6 | 7 | 8 | 9 | 10 |
| 13. I am able to follow a healthy eating plan when I am away from home | 0 | 1 | 2 | 3 | 4 | 5 | 6 | 7 | 8 | 9 | 10 |
| 14. I am able to choose different foods and maintain my eating plan when I am away from home | 0 | 1 | 2 | 3 | 4 | 5 | 6 | 7 | 8 | 9 | 10 |
| 15. I am able to follow a healthy eating plan during festive periods | 0 | 1 | 2 | 3 | 4 | 5 | 6 | 7 | 8 | 9 | 10 |
| 16. I am able to choose different foods and maintain a healthy eating plan when I am eating out or at a party | 0 | 1 | 2 | 3 | 4 | 5 | 6 | 7 | 8 | 9 | 10 |
| 17. I am able to maintain my eating plan when I am feeling stressed or anxious | 0 | 1 | 2 | 3 | 4 | 5 | 6 | 7 | 8 | 9 | 10 |
| 18. I am able to visit my doctor four times a year to monitor my diabetes Variance explained (%) | 0 | 1 | 2 | 3 | 4 | 5 | 6 | 7 | 8 | 9 | 10 |
| 19. I am able to take my medication as prescribed | 0 | 1 | 2 | 3 | 4 | 5 | 6 | 7 | 8 | 9 | 10 |
| 20. I am able to maintain my medication when I am ill | 0 | 1 | 2 | 3 | 4 | 5 | 6 | 7 | 8 | 9 | 10 |
