# Supplementary material for: Effects of Cognitive Behavioral Therapy for Diet on Postprandial Glucose and Pregnancy Outcomes in Gestational Diabetes Mellitus: Multicenter Randomized Controlled Trial
Source: J Med Internet Res. 2025 Jul 29;27:e71075. doi: 10.2196/71075 (PMC12306952; doi:10.2196/71075)
Supplement: Multimedia Appendix 4 [file jmir-v27-e71075-s004.docx]

After adjusting for baseline GQR at follow-up 1 (week 1–2) using ANCOVA, the intervention group demonstrated a significantly higher GQR at follow-up 3 (week 5–6) compared with the control group (mean difference: 4.0%, 95% CI: –8.0% to 0; P = 0.049). Although group differences at later time points did not reach statistical significance, the intervention group consistently showed numerically higher adjusted GQR values throughout the follow-up period (Table S1).

**Table S1.** Adjusted Between-Group Differences in GQR at Each Follow-up Time Point Based on ANCOVA.

| Follow-up  Time Point | Adjusted Mean (Control) (%) | Adjusted Mean (Intervention) (%) | Mean Difference (95% CI) | *P* value |
| --- | --- | --- | --- | --- |
| follow-up 2  (3-4 week) | 85.8% | 84.9% | -0.9% (-2.8 to 4.7) | 0.616 |
| follow-up 3  (5-6 week) | 82.9% | 86.9% | 4% (-8.0 to 0) | 0.049 |
| follow-up 4  (7-8 week) | 87.8% | 90.5% | 2.7% (-6.2 to 0.7) | 0.112 |
| follow-up 5  (9-10 week) | 91.7% | 93.8% | 2.1% (-4.6 to 0.4) | 0.097 |
| follow-up 6  (11-12 week) | 92.1% | 94.0% | 1.9% (-4.2 to 0.3) | 0.094 |

*Note:* Adjusted means and between-group differences in GQR were shown for each follow-up time point (follow-up 2 to 6). Values were derived from analysis of covariance (ANCOVA) models controlling for baseline GQR (follow-up 1). Adjusted means were estimated marginal means. Differences were presented as the absolute percentage point difference (Intervention-Control) with 95% confidence intervals. A positive value indicates a higher GQR in the intervention group.
